# The effect of auditor’s cooperation with independent financial advisors on the reliability of performance commitment—Based on the moderating effect of managerial overconfidence

**DOI:** 10.1371/journal.pone.0283125

**Published:** 2023-10-26

**Authors:** Dongchuan Lin, Yeling Wang, Qiqi Zheng, Hongyi Li

**Affiliations:** 1 School of Business and Tourism, Sichuan Agricultural University, Sichuan, China; 2 School of Accounting, Southwest University of Finance and Economics, Sichuan, China; University of Almeria: Universidad de Almeria, SPAIN

## Abstract

Based on the sample of major asset restructuring transactions of Shanghai and Shenzhen A-share listed companies from 2009 to 2018, this paper uses Logit model to examine the impact of auditors ’ cooperation experience with independent financial advisors on the reliability of performance commitments, and to examine the moderating effect of management overconfidence on the relationship between the two. The results show that the cooperation experience between auditors and independent financial advisors is significantly positively correlated with the reliability of performance commitment, and the closer the cooperation experience is, the easier the performance commitment will be realized, this has played a financial intermediary team effect. Further analysis shows that in the context of management overconfidence, the positive relationship between financial intermediaries ’ cooperation experience and the reliability of performance commitment is stronger. The research results enrich the research on the influencing factors of M & A performance commitment and the economic consequences of financial intermediaries ’ cooperation, and help the transaction subjects to rationally view performance commitment and regulators to improve policies and regulations.

## 1. Introduction

Along with the implementation of ’ the Belt and Road ’ strategy, China ’s listed companies through mergers and acquisitions(M&A) to achieve ’ going out ’ pace has not stopped, mergers and acquisitions activities also accompanied by frequent huge risks. According to statistics, nearly 40% of mergers and acquisitions in 2018 ended in failure. The performance commitment system can not only disperse the risk in the transaction process, solve the information gap between the two sides of the transaction, so that enterprises can obtain positive M & A benefits, but also force the improvement of M & A policy to ensure the stable operation of the capital market. China Securities Regulatory Commission (CSRC) issued in 2008 ’ major asset restructuring of listed companies management approach ’ that listed companies should issue a performance commitment agreement and audited by professional financial intermediaries. Performance commitment is essentially a future performance commitment made by the shareholders of the target company to the acquiring company under the condition of information asymmetry [1]. Although some mandatory provisions were removed in 2014, however, due to the high degree of gaming in M&A transactions, the acquirer protects its interests, the merged party transmits favorable information to the market, and they still tend to sign performance commitment agreements [2]. It has been suggested that the performance commitment agreement is beneficial to the synergy of M&A, greatly reduces the costs of acquirers through performance compensation [3], and it has an incentive effect on the performance of the target company after the merger [4]. But in terms of performance, it has the phenomenon of "high commitment", "high premium" and "low fulfillment" [5], in order to obtain equity incentives, the target company even adopts the method of earnings management to achieve "stepping on the line" performance standards [6], There is no doubt that it damages the interests of the acquirer and runs counter to the original intention of establishing a performance commitment.

Financial intermediaries play an independent supervisory role in M & A transactions and have a positive impact on the realization of performance commitments. Studies have shown that financial intermediaries can help reduce the search cost of the acquirer in the process of information acquisition and the cost of trial and error in the integration process [7]. Independent financial advisors are looking for high-quality targets [8], help enterprises negotiate and reduce communication costs [9], and M&A synergy to create M&A value for the acquirer [10]. When independent financial advisors are hired from the same place as the target company, their information advantage enhances the reliability of performance commitments [11]. Auditing of financial statements by auditors helps to improve the quality of accounting information [12], thereby reducing the possibility of failure on the way to M&A. However, financial intermediaries may also have ’ contradictory ’ psychology when providing services. For example, auditors often choose between customer requirements and professional obligations. On the one hand, professional standards require auditors to maintain complete independence, and on the other hand, auditors have conflicts of interest in their relationships with customers [13]. Auditors with higher professional knowledge can alleviate this situation in decision-making, because auditors with higher education will regard ’ independence ’ as the key to professional responsibilities, thus resisting customer pressure [14]. At the same time, when auditors have industry specialization capabilities, they can be promoted to implement adequate audit procedures, thereby improving audit quality [15]. In M & A transactions, shareholders will largely listen to financial advisers and may support the transaction [16]; The acquirer will also require replacement because of the low degree of specialization of the auditor [17]. And hiring expert consultants in the target industry is the most powerful way to obtain more superior information about the target company, thereby reducing the risk of overestimating the target value when improving M & A consulting services, formulating M & A plans, and determining transaction pricing [18].

Further, the auditor team can play a more positive role. The group theory holds that the construction of the auditor team helps to strengthen the psychological perception of members and reduce the communication cost between the two sides [19]. When the audit committee has cooperation experience with the auditor, it can promote mutual communication, information sharing, strengthen audit supervision, and improve audit independence [20]; The reputation theory holds that intermediaries pay more attention to long-term interests, and auditors will be cautious and spend more efforts to implement more audit procedures to improve audit quality. If both parties share an auditor, it will significantly improve the success rate of mergers and acquisitions [21]. Collusion theory holds that in an information asymmetry environment, market players will form a collusion relationship based on the maximization of personal interests. Financial intermediaries, which rely mainly on their clients ’ income to maintain their operations, may have an impact on independence [22] and ultimately harm the interests of minority shareholders, as the survey reports issued are targeted at external investors. However, the human capital of financial intermediaries, such as industry expertise, has an inhibitory effect on collusion [23]. The regulatory authorities also have strict supervision over financial intermediaries. If they do not comply with regulatory rules, they may be subject to administrative penalties, which will affect the reputation of the institution and ultimately lead to the loss of customers and even the normal operation of intermediaries. Therefore, financial intermediaries can strengthen independence through cooperation, achieve information sharing between auditors and independent financial advisers, and enhance the value of both parties by providing professional services.

In the existing literature, the research on the economic consequences of financial intermediaries mainly focuses on the impact on the quality of accounting information, mergers and acquisitions, investment and financing, Intermediaries include auditors, independent financial advisers and analysts. With the introduction of the CSRC ’s regulatory measures on mergers and acquisitions of listed companies, the number of mergers and acquisitions involving intermediaries such as auditors and independent financial advisers is increasing, and literature has begun to pay attention to their performance commitment agreements signed in mergers and acquisitions and the impact of whether the performance commitments are completed, but they are all based on a single auditor or a single independent financial adviser, lacking research from the perspective of financial intermediary cooperation. Specifically, the cooperation experience between auditors and independent financial advisers refers to whether there was cooperation between auditors and independent financial advisers before the M & A transaction; the reliability of performance commitment means that the cumulative actual performance of the target enterprise is greater than the promised performance during the performance commitment period.

In marketing activities, neuromarketing can provide value information related to consumer behavior, and make decisions by observing the response of consumers ’ brain regions when stimulated [24]. New technologies such as functional magnetic resonance imaging and electroencephalogram are used to understand consumer behavior and cognitive processes, but traditional methods are difficult to achieve [25] Therefore, the characterization of individual psychological behavior needs to rely on new technologies and new methods to measure. The management has a higher position in the enterprise, irrational M & A decisions will have a great impact on enterprises. Behavioral finance theory holds that overconfident management is more likely to implement M & A activities, because people with irrational behavior characteristics tend to be blindly optimistic about M & A transactions and underestimate M & A risks [26]; it will also overestimate its ability to control market risks. In order to build a personal business empire, it is willing to pay a higher price than the assessed value of the merged enterprise, forming a higher M & A premium [27]; at the same time, it is overly optimistic about the operating conditions and performance capabilities of the target companies, leading to high commitments and increasing the risk of performance loss after mergers and acquisitions [28].On the contrary, if the acquirer shows accounting conservatism, it can increase the possibility of achieving the target commitment by reducing the commitment standard of the target company and increasing the post-merger supervision of the target company [29]. The feasibility opinions issued by independent financial advisers can enable stakeholders to fully understand the operating conditions [28]. On the research of management overconfidence in M & A transactions, the existing literature mainly focuses on the value level of M & A transactions, such as M & A performance, M & A premium, and studies its economic consequences from the internal perspective of single management overconfidence. Financial intermediaries can restrain the irrational behavior of managers and reduce losses [30]. The choice and behavior of external financial intermediaries will be affected by the management. Therefore, the management characteristics is also considered in this paper. We takes management overconfidence as a moderating variable to examine whether the external financial intermediary effect is heterogeneous owing to different management characteristics.

In view of this, this paper selects the major asset restructuring transactions of Shanghai and Shenzhen A-share listed companies as acquirers from 2009 to 2018 as samples to study the impact of the cooperation experience of auditors and independent financial advisers on the reliability of performance commitments, and the internal factor of management overconfidence is used as a moderator variable to test whether it plays a regulatory role. This paper mainly uses logit regression model to conduct descriptive analysis, correlation analysis, t-test and logit regression on sample objects, and conducts robustness tests by replacing variable measurement methods, regression models and endogenous tests. The research results are as follows: The previous relationship between auditors and independent financial advisers helps to alleviate the information weakness of a single subject, enhance the rationality of performance commitment agreements and urge performance commitments to be fulfilled, thereby improving the reliability of performance commitments; This positive correlation is more obvious when the management of the acquirer is overconfident, which means that the cooperation of financial intermediaries can inhibit the low information quality problem caused by the management overconfidence.This means that the cooperation experience of auditors and independent financial advisers can play a team effect, which has a positive impact on the pre-audit, in-process transaction supervision and post-resource integration of M & A transactions, and jointly restrain the irrational behavior of management, and finally achieve better M & A performance under the performance commitment system.

The main contributions are as follows: (1) Existing studies mostly discuss the governance effect from the perspective of single subject of financial intermediaries such as auditors or independent financial advisers. This paper studies the impact of the cooperative relationship between auditors and independent financial advisers on the reliability of performance commitments from the perspective of team effect, which expands the application scenarios of team theory. (2)This paper not only considers the governance effect of external financial intermediaries, but also takes into account the irrational behavior characteristics of internal management, and enriches the research on the influencing factors of the reliability of performance commitment from the perspective of internal and external governance effects. (3) The research results of this paper provide a policy basis for the CSRC to improve the performance commitment system, how to choose financial intermediaries in M & A practice, and how financial intermediaries themselves function.

## 2. Theoretical analysis and research hypotheses

### 2.1 Financial intermediary cooperation and reliability of performance commitment

The CSRC explicitly requires that accounting firms and securities service agencies be hired to provide consulting services in major asset restructuring transactions. The core business of auditors is to audit financial statements, and the information transparency after auditing is higher [31]; Independent financial advisors screen out valuable target enterprises through due diligence [10], helping to design a reasonable M&A plan. When people who share common interests are organized and pursue the same goal, they form a group. In M&A, team theory believes that the energy of financial intermediaries is limited, and independent financial advisers usually choose cooperative accounting firms, so that communication efficiency will be greatly improved. Therefore, accounting firms tend to share real accounting information with securities companies to achieve long-term cooperation [32].the team effect formed between financial intermediaries can play an important role. Reasonable performance commitment is the premise to ensure the effective implementation of performance commitment agreements and reduce the risk of mergers and acquisitions [33]. If the auditor and the independent financial adviser form a cooperative relationship, the team advantage can be brought into play.On the one hand, in the stage of formulating performance commitments, financial intermediary cooperation can give full play to its advantages in financial tools, market accumulation and other aspects, assist M & A parties to complete transaction information review, select appropriate target enterprises, design M & A plans, reduce the uncertainty in M & A, and achieve high-quality M & A [34]; we should carefully evaluate the value of the merged party, formulate reasonable performance commitment goals and follow-up performance safeguards [35], make the implementation of M & A transactions more reasonable and efficient, and avoid excessive payment risks [36].From the perspective of co-audit, co-auditors can enable acquirers to obtain more implicit information and provide a basis for evaluating the performance of the target company [21]. At the same time, acquirers have stronger premium ability than other investors, so that the reliability of performance commitments will be higher.And in the implementation stage of mergers and acquisitions, financial intermediaries use existing information to propose reasonable M & A transaction consideration, reduce the high premium risk of mergers and acquisitions, and ensure that the target company obtains asset returns.On the other hand, in the performance commitment implementation stage, the cooperative relationship between financial intermediaries has a stronger supervision on the performance commitment implementation of the merged party, reduces the opportunistic behavior of the target enterprise, and avoids the completion of performance commitments by inflating profits and reducing costs [37].Therefore, regardless of the stage of M & A, the cooperative relationship of financial intermediaries can give full play to their advantages, thereby improving the reliability of performance commitments [18].

The theory of collusion was first proposed in 1929 by the economist Carl Cattell and applied to competitive market environments, arguing that market players will form a collusion relationship with each other in order to obtain benefits. In 1993, Laffont applied this theory to the environment of asymmetric information. M & a transaction completion hypothesis that financial intermediaries provide services only to obtain commissions and despise the interests of both sides of the merger, in the process of mergers and acquisitions show that the target company audit and investigation is not strict [38]. Accordingly, financial intermediaries may form a conspiracy relationship based on business dependence and cost-benefit trade-offs [39] prompting financial intermediaries to pay more attention to the volume of M & A transactions rather than the quality of M & A transactions, and to make no contribution to the reasonableness of performance commitments, resulting in the frequent occurrence of unreasonable high M & A consideration of the target company, thereby increasing the probability of performance failure after M & A.

At present, accounting firms and securities service institutions show the characteristics of large volume in the market. The CSRC also implements diversified assessments for financial intermediaries and is more stringent. According to this, various financial institutions are facing high competitive pressure and high risks. If auditors and independent financial advisers pay attention to mutual cooperation in transactions and promote the completion of transactions based on the advantages of knowledge complementarity and information sharing, it not only improves the efficiency of transaction completion, but also helps financial institutions on both sides to enhance their competitiveness. In addition, due to China ’s capital market is relatively perfect, auditors and independent financial advisers in mergers and acquisitions transactions will participate in each link, through due diligence to find valuable target companies before mergers and acquisitions, mergers and acquisitions to fully consider the rationality of the commitment of the target companies, as well as the integration of resources on both sides after the merger, and the China Securities Regulatory Commission clearly stipulates that intermediaries to fulfill the obligation of continuous supervision, if the actual profit does not reach a certain proportion will be subject to regulatory talks, issued a warning letter and other regulatory measures, thus affecting the reputation of institutions. Therefore, in the fierce market competition environment, financial intermediaries tend to cooperate based on the consideration of enhancing their competitiveness and supervision. Based on this, this paper expects that auditors and independent financial advisers are more likely to have a cooperative effect rather than a collusion effect, and then puts forward hypothesis one:

H1: The cooperation experience between auditors and independent financial advisers significantly improves the reliability of performance commitments.

### 2.2 The mechanism of management’s overconfidence

Overconfidence is a manifestation of self-deviation in psychology. Behavioral finance theory holds that overconfident managers will exaggerate their abilities and believe that investment decisions will produce positive results. Even if they fail, managers will think that it is the environment [40]. Overconfident managers will promote overinvestment and reduce investment efficiency [41]; in this process, management is more inclined to use operating cash flow investment rather than stocks or bonds, and shows greater manipulation of operating cash flow than rational peer companies [42].Overconfident managers tend to overestimate their own capabilities, underestimate external risks, tend to large-scale M&A, and overestimate the control of M&A risks [43]; Overconfident management will also promote frequent and inefficient mergers and acquisitions, and ultimately pay excessive M & A premiums [44]; the more serious the overconfidence is, the higher the M & A premium it pays in M & A activities [45]. At the same time, the implementation of earnings management in order to achieve the target performance results in a gradual decline in the performance of post-merger acquisitions [46]. Auditing standards stipulate that understanding customer managers is part of the auditor ’s risk assessment process, and overconfident managers may propose higher-risk plans, resulting in uncertain results, thereby increasing the likelihood that the company will receive going-concern opinions [47]. At the same time, overconfident management is more likely to issue more optimistic earnings forecasts than real earnings [48]; rational management will follow the advice of more conservative target advisors [16].

In major asset restructuring transactions, overconfident management will be too optimistic to estimate the profitability of the target company, while ignoring the possible risks and the false high level of performance commitment of the target company, but independent financial advisers can significantly suppress the negative impact of management overconfidence [28].On the one hand, financial intermediaries will use the information transmission mechanism to realize information sharing in order to complete M&A transactions, stop decisions that damage the interests of the company in a timely manner, and play a role in supervising management’s decision-making [49]; On the other hand, financial intermediaries are responsible for the business performed, and face greater risks in selecting target companies, pre-acquisition negotiation, and evaluation, and post-acquisition integration [50], when earnings forecast actual profits if it does not reach more than 80%, the CSRC requires an apology to shareholders and public investors, and even more administrative penalties from the CSRC. Therefore, financial intermediaries will consider the completion of M&A transactions and risk factors and focus on independence in providing services, and the performance commitment value determined by the target company is more reasonable. Strengthen cooperation between financial intermediaries, jointly provide high-quality consulting services to M&A, and support the development of post-merger synergies. Therefore, when the management is overconfident, financial intermediaries are more inclined to cooperate based on risk and reputation, play a team effect and improve the reliability of performance commitment.

Therefore, this paper proposes the following competing hypothesis two:

H2: In the context of management overconfidence, the experience of auditors working with independent financial advisors increases the reliability of performance commitments more significantly.

## 3. Research design

### 3.1 Data

The research object of this paper was focused on the listed companies that initiated major asset restructuring transactions and signed performance commitment agreements from 2009 to 2018.Finally, there are 197 companies and 584 sample data.The specific data screening process is as follows: (1) Since the definition of financial intermediary cooperation relies on established sample data, only the earliest merger in the year of a merger is reserved for multiple M&A initiated by the same listed company; (2) Exclude financial, ST, and *ST listed companies; (3) Eliminate M&A whose purpose is backdoor listing; (4) Eliminate incomplete transaction information and missing samples.

All continuous variables are Winsorized at the upper and lower ends of 1%. M&A transaction data and financial data are from Wind, CSMAR, And through manual collection and verification from official websites such as www.cninfo.com.cn and Shenzhen Stock Exchange. Data processing adopts Stata15.0 and Excel software.

### 3.2 Definition of variables

#### 3.2.1. Reliability of performance commitments

This paper uses the difference between actual performance and commitment performance to measure the reliability of performance commitment. If the actual performance is greater than the commitment performance, it indicates that the performance commitment is reliable. Draws on the measurement methods of Dou Wei [28] and Yu Yumiao [51], using the form of dummy variables. If in the performance commitment period, each accounting year ’ s actual profit is greater than or equal to the promised profit is 1, otherwise 0. At the same time, in the robustness test section, the ratio of the actual performance of the target company to the promised performance is used to measure the reliability of the performance commitment [51]. The continuous variable used at this time can make up for the deficiency of the dummy variable. The larger the value is, the better the performance commitment of the target company is completed. When the value is greater than 1, it indicates that the performance commitment of the target company is completed.

#### 3.2.2. Auditor and independent financial adviser cooperation experience

In this paper, whether there is cooperation between auditors and independent financial advisers before this major asset restructuring transaction is used to represent financial intermediary cooperation (Coop1). If there is, it is defined as 1, otherwise it is 0. This is because the CSRC clearly requires financial intermediaries to provide services to M & A transactions, so the accumulation of similar businesses is crucial. If auditors and independent financial advisors have worked together before, then the quality of communication in the course of the transaction will be improved, and the two are likely to generate knowledge spillovers based on previous cooperation, information sharing, which helps to improve the quality of service. At the same time, when one party has more information and industry expertise, the other party can have a learning effect, increasing the likelihood of a successful transaction. In addition, this paper defines financial intermediary cooperation (Coop2) based on the number of times auditors cooperate with independent financial advisers, which is expressed by the natural logarithm of the number of times auditors cooperate with independent financial advisers plus one.

#### 3.2.3. Overconfident management

Because overconfidence belongs to the category of psychology, it is difficult to measure accurately. In the existing literature, it is mainly indirectly measured from the reflection of the behavior results of executives on the financial characteristics of the company, including the relative compensation of executives (Jiang Fuxiu et al., 2009) [52], the accuracy of performance forecast (Yu Minggui et al., 2006), the change of shareholding (Hao Ying et al., 2005) [53]. Therefore, this paper uses the most widely used alternative indicator in the academic community-the relative compensation of executives to measure. Relative executive pay is expressed as the ratio of the sum of the top three highest paid executives to the sum of all executives, the higher the relative salary of the management, the higher the value of the management is recognized by the enterprise, which also reflects the relative importance of the manager in the company, and then makes overly optimistic management decisions. Therefore, the larger the index value, the more confident the management. In the robustness test section, this paper selects the accuracy of earnings forecast (YuMinggui, 2006) to measure management overconfidence.

#### 3.2.4. Control variables

In addition, this paper refers to the research of Dou Wei and Wu Hengguang to control the variables that may affect the reliability of performance commitment, it mainly includes company-level characteristic variables such as asset-liability ratio, return on equity, actual controller nature, Tobin ’s Q value, institutional investor shareholding ratio, investment opportunities, equity concentration, market-to-book ratio, etc. and associated attributes, compensation direction, compensation M & A transaction level characteristic variables. See S1 Appendix for variable definitions.

### 3.3 Model

This paper constructs the following model to test the hypothesis:

$$RVAM=\beta_{0}+\beta_{1}Coop1/Coop2+\beta_{2}Control+\varepsilon$$ (1)

$$RVAM=\beta 0+\beta_{1}Coop1/Coop2+\beta_{2}OC+\beta_{3}Coop1*OC+\beta_{4}Control+\varepsilon$$ (2)

## 4. Empirical results and analysis

### 4.1 Descriptive statistical analysis

Table 1 lists the descriptive statistics of the 583 sample observations in this paper. The mean value of RVAM is 0.648, indicating that 64.8% of the target companies have completed their performance commitments within the commitment period, But about 1/3 of the companies did not meet the performance standards during the commitment period, it shows that the phenomenon of performance failure exists widely in practice. The mean value of Coop1 is 0.564, It shows that 56.4% of the acquirers in my country’s M&A and restructuring transactions tend to hire auditors and independent financial advisors who have cooperated before; The mean value of Coop2 is 1.195, indicating that the auditors and independent financial advisors have a higher degree of cooperation in the sample. The mean of OC is 0.499, which indicates that management has a high degree of overconfidence. The mean value of Ownership is 0.268, indicating that only 26.8% of the samples are state-owned enterprises, This indicates that major assets reorganization transactions are concentrated in non-state-owned enterprises, which may be due to the complexity of state-owned enterprise M&A process and high time cost. The mean value of TVAM is 0.739, indicating that 73.9% of the samples adopt non-cash compensation methods, It shows that the enterprises winning the bid in China’s M&A transaction market are better at using equity or the compensation method of equity and cash. Other descriptive results were broadly consistent with existing research.

**Table 1:** Descriptive statistics results.

Variable	N	mean	sd	min	max
RVAM	583	0.648	0.478	0	1
Coop1	583	0.564	0.496	0	1
Coop2	583	1.195	0.573	0.693	3.045
OC	583	0.499	0.500	0	1
TVAM	583	0.739	0.439	0	1
DVAM	583	0.254	0.436	0	1
Relate	583	0.691	0.462	0	1
Lev	583	0.382	0.204	0.0446	0.886
ROE	583	0.069	0.068	-0.192	0.330
TobinQ	583	2.508	1.547	0.994	10.064
Ownership	583	0.268	0.443	0	1
Institute	583	40.750	21.023	0.971	86.262
Growth	583	0.525	0.961	-0.567	6.444
EC	583	0.058	0.077	0.000	0.394
MB	583	0.590	0.574	0.079	3.861

### 4.2 Correlation analysis

Table 2 is the correlation analysis results of variables. There is a significant positive correlation between RVAM and Coop1 at the level of 5%, and the correlation coefficient is 0.106, indicating that the experience of auditors working with independent financial advisers helps to improve the reliability of performance commitments. TVAM, Relate, Lev, TobinQ, ownership, and institute have significant correlations with RVAM. In addition, in order to verify whether there is a multicollinearity problem between variables, the VIF test is carried out in this paper. The mean VIF is 1.23, which is far less than 5, which shows that there is no collinearity problem between variables and can be further analyzed.

**Table 2:** Correlation analysis.

Variables	(1)	(2)	(3)	(4)	(5)	(6)	(7)	(8)	(9)	(10)	(11)	(12)	(13)
(1) RVAM	1.000												
(2) Coop1	0.106**	1.000											
(3) TVAM	0.086**	0.038	1.000										
(4) DVAM	0.000	0.099**	0.086**	1.000									
(5) Relate	0.099**	-0.019	0.054	-0.345***	1.000								
(6) Lev	0.096**	-0.031	-0.017	-0.120***	0.199***	1.000							
(7) ROE	0.063	-0.041	-0.012	-0.115***	0.065	0.016	1.000						
(8) TobinQ	0.068*	0.021	-0.017	0.031	-0.062	-0.202***	0.090**	1.000					
(9) Ownership	0.112***	-0.063	0.006	-0.219***	0.292***	0.361***	0.026	-0.109***	1.000				
(10) Institute	0.092**	-0.020	-0.008	-0.189***	0.183***	0.232***	0.121***	0.006	0.292***	1.000			
(11) Growth	-0.038	0.051	0.047	-0.055	0.045	-0.015	0.134***	0.151***	-0.036	0.144***	1.000		
(12) EC	0.039	-0.058	-0.074*	-0.149***	0.143***	0.182***	0.009	-0.085**	0.347***	0.445***	-0.096**	1.000	
(13) MB	0.021	-0.051	-0.003	-0.122***	0.195***	0.480***	-0.044	-0.205***	0.277***	0.177***	0.076*	0.223***	1.000

*** p<0.01

** p<0.05

* p<0.1

### 4.3 T-test

Table 3 lists the t-test results of financial intermediary cooperation and cooperation degree grouped by the reliability of performance commitment. For the sample of performance commitment reliability, the mean value of financial intermediary cooperation is 0.60, For samples with unreliable performance commitments, the mean value of financial intermediary cooperation is 0.49, the T value is 2.652, the Z value is 2.567, at the level of 5% and 1% respectively, The difference between the two groups was statistically significant, this means that financial intermediary cooperation is more conducive to achieving the reliability of performance commitments. Similarly, for the degree of financial intermediary cooperation, the reliability of its performance commitment is significantly higher than that of the samples with unreliable performance commitments, at the level of 10% and 5% respectively. Hypothesis 1 is preliminarily verified, but the regression results need to be further verified.

**Table 3:** Independent sample T-test and Mann-Whitney U test.

	Reliable performance promises	Unreliable performance promises	T-test (|T|)	Mann-Whitney U test (|Z| value)
Variable	mean	Mean	H0: Unreliable H1: Reliable	H0: Unreliable H1: Reliable
Coop1	0.60	0.49	2.562**	2.567***
Coop2	1.224	1.141	1.678*	2.082**

## 4.4 Analysis of regression results

### 4.4.1 Financial intermediary cooperation and reliability of performance commitments

Table 4 shows the Logit regression results of financial intermediary cooperation on the reliability of performance commitments. Column (1) performs logit regression on financial intermediary cooperation and performance commitment reliability, the coefficient of Coop1 is significantly positive at the 1% level, That is, financial intermediary cooperation can help improve the reliability of performance commitments, this shows that auditors and independent financial advisors may play a team effect rather than a collusion effect based on the consideration of resource sharing, market expansion [20] and reputational motivation, Hypothesis one is verified; Furthermore, the coefficient of TVAM is significantly positive at the 10% level, explain that the listed company’s use of equity compensation or equity plus cash compensation is conducive to the realization of performance commitments; The coefficient of Ownership is also significantly positive at the 10% level, indicating that when the acquirer is state-owned, it helps to improve the reliability of performance commitments, which is due to the stricter supervision of state-owned enterprises. Column (2) performs logit regression on the degree of financial intermediary cooperation and the reliability of performance commitments, the coefficient of Coop2 is significantly positive at the 10% level, which means that the more the auditors cooperate with the independent financial adviser, the more helpful it is to achieve the performance commitment, the results of TVAM and Ownership are also consistent with Coop1, which further verifies the team governance effect of financial intermediary cooperation, that is, financial intermediary cooperation is conducive to the realization of performance commitments.

**Table 4:** Logit regression results of financial intermediary cooperation and performance commitment reliability.

Variable	RVAM	RVAM
Coop1	0.486***(2.68)	
Coop2		0.286*(1.73)
TVAM	0.389*(1.90)	0.400**(1.96)
DVAM	0.188(0.83)	0.196(0.87)
Relate	0.346(1.57)	0.351(1.60)
Lev	0.969*(1.76)	0.988*(1.80)
ROE	1.934(1.41)	1.857(1.37)
Tobin Q	0.136**(1.97)	0.127*(1.84)
Ownership	0.420*(1.72)	0.412*(1.70)
Institute	0.006(1.30)	0.007(1.36)
Growth	-0.164*(-1.66)	-0.152(-1.58)
EC	-0.439(-0.33)	-0.487(-0.37)
MB	-0.174(-0.98)	-0.189(-1.04)
Ind/Year	Control	control
Constant	-1.209***(-3.10)	-1.279***(-3.08)
N	583	583
Pseudo R2	0.0472	0.0420

#### 4.4.2 Financial intermediary cooperation, management overconfidence and reliability of performance commitments

Based on the above analysis results, we further explore the moderating effect of financial intermediary cooperation on the reliability of performance commitments in the context of managerial overconfidence. Table 5 presents the regression results grouped by management overconfidence. Columns (1) and (2) are the moderating effects of financial intermediary cooperation and performance commitment reliability, the Coop1 coefficient is significantly positive at the 1% level in the sample of management overconfidence, but not statistically significant in the sample without overconfidence. Columns (3) and (4) are the moderating effects between the degree of financial intermediary cooperation and the reliability of performance commitments, the coefficient of Coop2 is significantly positive at the 1% level in the sample of management overconfidence, but not statistically significant in the sample without overconfidence. Research shows that financial intermediaries are more inclined to cooperate to play a team effect and improve the reliability of performance commitments in the context of management overconfidence; The higher the degree of cooperation, the more pronounced this effect is. This shows that when the management is overconfident, financial intermediaries will investigate the information of listed companies and make more stringent decision-making based on reputation considerations, so that the performance commitment value of the target company is reasonable, thereby improving the reliability of performance commitments. Hypothesis two is verified.

**Table 5:** Results of the moderating effect of management overconfidence.

Variable	RVAM		RVAM	
	(1) OC = 1	(2) OC = 0	(3) OC = 1	(4) OC = 0
Coop1	0.827***(3.09)	0.224(0.83)		
Coop2			0.701***(2.71)	-0.084(-0.35)
TVAM	0.718**(2.39)	0.125(0.42)	0.743**(2.47)	0.153(0.51)
DVAM	-0.135(-0.39)	0.315(0.97)	-0.143(-0.42)	0.361(1.12)
Relate	0.579*(1.80)	0.237(0.75)	0.557*(1.75)	0.248(0.79)
Lev	1.941**(2.56)	-0.285(-0.33)	1.971***(2.62)	-0.233(-0.27)
ROE	-1.167(-0.53)	4.524**(2.36)	-1.158(-0.54)	4.413**(2.30)
TobinQ	0.125(1.48)	0.173(1.39)	0.102(1.19)	0.168(1.37)
Ownership	0.400(1.15)	0.409(1.14)	0.422(1.23)	0.376(1.06)
Institute	-0.005(-0.75)	0.018**(2.45)	-0.005(-0.65)	0.018**(2.43)
Growth	0.039(0.29)	-0.340**(-2.43)	0.047(0.35)	-0.328**(-2.43)
EC	0.617(0.32)	-1.799(-0.97)	0.320(0.17)	-1.621(-0.88)
MB	-0.310(-1.38)	0.020(0.07)	-0.287(-1.23)	-0.028(-0.10)
Ind/Year	control	control	control	control
Constant	-1.438**(-2.44)	-0.974(-1.61)	-1.803***(-2.84)	-0.767(-1.19)
N	291	292	291	292
Pseudo R2	0.0865	0.0663	0.0847	0.0647

### 4.5 Robustness test

#### 4.5.1 Measurement of substitution variables

*1*. *Replace the explained variable*. In this paper, when the actual performance of each accounting year is lower than the promised performance, it is defined as the unreliable performance commitment, that is, the dummy variable. However, due to the different completion of each enterprise, it is more realistic to measure the reliability of performance commitment by the completion of the actual performance of the enterprise, which is embodied in the ratio of actual performance to commitment performance during the commitment period. After re-collecting the data, the regression test was carried out again. The results showed that the correlation coefficient of Coop1 was 9.343, showing a significant positive correlation at the 1% level, indicating that the auditor ’s experience with independent financial advisers helped to improve the reliability of performance commitments and verified the reliability of the conclusions of this paper. in the context of management overconfidence, still consistent with the above results, the regression results are robust. The details are shown in Table 6.

**Table 6:** Financial intermediary cooperation and performance commitment reliability.

Variable	RVAM		RVAM	
	(1)	(2)	(3) OC = 1	(4) OC = 0
Coop1	9.343***(2.67)		15.166***(2.95)	4.410(0.93)
Coop2		4.314(1.42)		
TVAM	6.554*(1.77)	6.504*(1.75)	4.788(0.89)	8.885*(1.76)
DVAM	-2.221(-0.55)	-2.127(-0.53)	-1.546(-0.25)	-3.462(-0.64)
Relate	0.896(0.23)	1.192(0.30)	7.395(1.25)	-4.149(-0.77)
Lev	-9.488(-0.93)	-8.720(-0.85)	-20.781(-1.45)	12.602(0.84)
ROE	39.063**(2.35)	39.302**(2.35)	15.674(0.68)	62.033**(2.36)
TobinQ	-1.616(-1.39)	-1.722(-1.47)	-0.500(-0.32)	-2.137(-1.15)
Ownership	8.683**(2.02)	8.974**(2.08)	9.208(1.45)	12.502*(1.94)
Institute	-0.147(-1.62)	-0.145(-1.58)	-0.202(-1.48)	-0.105(-0.84)
Growth	-1.752(-0.99)	-1.564(-0.88)	-1.376(-0.54)	-1.485(-0.59)
EC	44.446*(1.79)	43.179*(1.73)	36.382(0.99)	29.444(0.88)
MB	10.567***(2.87)	10.475***(2.83)	19.376***(3.85)	-5.473(-0.96)
Ind/Year	control	control	control	control
Constant	94.565***(4.72)	90.321***(4.46)	94.151***(3.70)	38.491(1.07)
N	583	583	291	292
R-squared	0.120	0.111	0.222	0.231
adj_ R2	0.0583	0.0494	0.105	0.115
F	1.948	1.796	1.892	1.995

*2*. *Replacing the adjustment variable*. In the existing studies, the replacement indicators of management overconfidence are complex and diverse, such as the relative compensation of executives of Jiang Fuxiu et al. (2009), the accuracy of the performance forecast of Yu Minggui et al. (2006), the change of the shareholding quantity of Hao Ying et al. (2005) [53]. The previous article refers to the method of Jiang Fuxiu et al. (2009), Although it has been widely used in the academic world, in order to avoid measurement deviation caused by the selection of measurement indicators, this paper replaces and verifies the indicators of management overconfidence. The performance forecast is made by the management before the actual performance is officially disclosed, which can better show the management’s subjective expectations of the company, this paper selects the measurement method of Yu Minggui [54], when the performance forecast is made by the management is higher than the actual performance, it is defined as overconfidence, and it is assigned a value of 1, otherwise it is 0. The accuracy of the performance forecast was used as a moderating variable for grouping regression, and the results showed that it was significantly positive in the context of management overconfidence, and the regression results were consistent with the previous research results. As shown in Table 7.

**Table 7:** Results of the moderating effect of management overconfidence.

variable	RVAM		RVAM	
	(1) OC = 1	(2) OC = 0	(3) OC = 1	(4) OC = 0
Coop1	0.728**(2.24)	0.404*(1.81)		
Coop2			0.571*(1.93)	0.162(0.80)
TVAM	0.024(0.06)	0.503**(2.00)	0.037(0.10)	0.507**(2.03)
DVAM	0.408(0.98)	0.095(0.34)	0.350(0.84)	0.125(0.45)
Relate	0.593(1.54)	0.244(0.90)	0.592(1.54)	0.256(0.95)
Lev	0.397(0.42)	1.287*(1.84)	0.486(0.51)	1.278*(1.85)
ROE	1.808(0.85)	1.980(1.04)	1.514(0.74)	2.006(1.05)
TobinQ	0.164(1.57)	0.126(1.36)	0.146(1.39)	0.125(1.36)
Ownership	0.262(0.61)	0.505(1.64)	0.295(0.70)	0.476(1.55)
Institute	0.004(0.43)	0.007(1.20)	0.004(0.49)	0.008(1.28)
Growth	-0.334**(-2.18)	-0.033(-0.23)	-0.305**(-2.01)	-0.032(-0.23)
EC	-0.857(-0.40)	-0.379(-0.21)	-0.880(-0.42)	-0.482(-0.27)
MB	0.093(0.35)	-0.338(-1.26)	0.071(0.27)	-0.346(-1.24)
Ind/Year	control	control	control	control
Constant	-0.911(-1.31)	-1.330***(-2.70)	-1.176(-1.61)	-1.319**(-2.56)
N	207	376	207	376

#### 4.5.2 Retest with Probit model

The Logit model used in this paper is a cumulative distribution function that conforms to the "logical distribution", and the residual distribution is non-normal, however, in order to test the robustness of the results in this paper, the Probit model is used to re-estimate the sample, The results of the study are shown in Table 8. Columns (1) and (2) are the estimation results of the reliability of performance commitment of financial intermediary cooperation. The results show that the coefficients of Coop1 and Coop2 are significantly positive at least 10%, which verifies the previous research results. Columns (3), (4), (5) and (6) are the regulatory mechanisms to test management overconfidence. The results show that Coop1 and Coop2 coefficients are significantly positive at the 1% level, which confirms the robustness of the conclusions in this paper.

**Table 8:** Probit model regression results.

variable	RVAM		RVAM		RVAM	
	(1)	(2)	(3) OC = 1	(4) OC = 0	(5) OC = 1	(6) OC = 0
Coop1	0.301***		0.498***	0.133		
	(2.73)		(3.12)	(0.82)		
Coop2		0.176*			0.412***	-0.052
		(1.78)			(2.79)	(-0.36)
TVAM	0.238*	0.245**	0.427**	0.068	0.444**	0.083
	(1.91)	(1.97)	(2.39)	(0.38)	(2.47)	(0.46)
DVAM	0.114	0.118	-0.069	0.196	-0.078	0.225
	(0.83)	(0.86)	(-0.34)	(1.01)	(-0.38)	(1.16)
Relate	0.216	0.219	0.347*	0.145	0.332*	0.152
	(1.61)	(1.64)	(1.81)	(0.75)	(1.74)	(0.79)
Lev	0.577*	0.585*	1.139**	-0.194	1.158***	-0.173
	(1.74)	(1.77)	(2.56)	(-0.37)	(2.61)	(-0.33)
ROE	1.193	1.144	-0.586	2.826**	-0.624	2.755**
	(1.44)	(1.39)	(-0.45)	(2.42)	(-0.50)	(2.37)
TobinQ	0.080**	0.074*	0.076	0.099	0.061	0.097
	(2.02)	(1.87)	(1.54)	(1.43)	(1.23)	(1.41)
Ownership	0.255*	0.249*	0.238	0.252	0.263	0.231
	(1.74)	(1.71)	(1.13)	(1.17)	(1.27)	(1.09)
Institute	0.004	0.004	-0.003	0.011**	-0.002	0.011**
	(1.28)	(1.37)	(-0.70)	(2.48)	(-0.56)	(2.47)
Growth	-0.100*	-0.092	0.018	-0.208**	0.025	-0.202**
	(-1.67)	(-1.56)	(0.21)	(-2.44)	(0.30)	(-2.43)
EC	-0.284	-0.311	0.340	-1.096	0.144	-1.000
	(-0.35)	(-0.38)	(0.29)	(-0.97)	(0.12)	(-0.88)
MB	-0.103	-0.112	-0.183	0.018	-0.166	-0.006
	(-0.93)	(-1.00)	(-1.29)	(0.10)	(-1.14)	(-0.04)
Constant	-0.729***	-0.772***	-0.861**	-0.573	-1.075***	-0.445
	(-3.09)	(-3.07)	(-2.47)	(-1.62)	(-2.89)	(-1.17)
N	583	583	291	292	291	292

#### 4.5.3 Propensity score matching

In order to avoid the influence of sample selection bias on the research results, this paper uses propensity score matching (PSM) to test for endogeneity. According to model (1), asset-liability ratio, market-to-book ratio, institutional investor shareholding ratio, equity concentration, Tobin’s Q value, nature of actual controller, related attributes and compensation method are used as matching factors, and Coop1 is used as processing variable PSM one-to-one matching for all samples, after excluding 2 pieces of data that are not in the common value range, 581 observations of matching samples are obtained. Using the logit model to calculate the propensity score, the results show that the matched samples are still significantly positive at the 1% level, which is consistent with the previous results. As shown in Table 9.

**Table 9:** PSM results.

variable	RVAM
Coop1	0.624***(2.63)
TVAM	0.456*(1.74)
Relate	0.206(0.74)
Lev	1.005(1.42)
TobinQ	0.052(0.65)
Ownership	0.175(0.57)
Institute	0.000(0.05)
EC	0.472(0.27)
MB	-0.231(-0.99)
Ind/Year	control
Constant	-0.686(-1.48)
N	324

## 5. Conclusions and enlightenment

With the implementation of the ’ Belt and Road ’ strategy, China ’s listed companies mergers and acquisitions boom has not declined. auditors and independent financial advisers, as the ’ gatekeepers ’ of the capital market, have been given the responsibility of regulators. The quality of service provided in M & A transactions directly determines the value of the transaction. However, due to the inconsistent market functions of auditors and independent financial advisers, the information they have may be missing. The behavior of internal managers affects the decision-making of enterprises. If the management has overconfidence, it is easy to overestimate the ability of their own control and underestimate the risk, thus increasing the risk of mergers and acquisitions. Therefore, this paper examines the impact on the reliability of performance commitment by taking into account the behavior of external financial institutions and internal managers. The study found that the experience of auditors working with independent financial advisers helps to improve the reliability of performance commitments, and the degree of cooperation between the two also positively affects the reliability of performance commitments to a certain extent; It is further found that in the context of management overconfidence, the cooperation experience between auditors and independent financial advisers has a more significant impact on the reliability of performance commitments. Research shows that independent financial advisers can significantly improve the reliability of performance commitments of listed companies with management overconfidence, but the direct effect on alleviating the problem of unreliable performance commitments is not obvious, that is, to a certain extent, financial intermediaries have a positive effect. In this paper, the direct effect of the cooperation experience between auditors and independent financial advisors on the reliability of performance commitment is also positive, which shows that financial intermediaries themselves have better governance effect, and the effect of their cooperation is more obvious.

This paper also has shortcomings in measuring performance commitment and the cooperation experience between auditors and independent financial advisers. It mainly uses the measurement method of dummy variables, which may lead to the generalization of research conclusions. Therefore, follow-up research on financial intermediation or performance commitment can continue to improve the measurement indicators. Define weights according to business revenue, number of customers, or the size of performance commitments, and then construct an index based on each weight, so as to measure by adopting indicators of continuous variables, which can make the research conclusions more feasible and universal.

The enlightenment of this paper is that financial intermediary cooperation, as a typical informal system design, can effectively alleviate the phenomenon of " performance face change " in the capital market and improve the probability of achieving performance commitments. In practice, under the guidance of the formal system, the acquirer can prevent financial risks from the perspective of the informal system, but also consider the market environment in which they are located. When signing performance commitments, they must not blindly believe in high commitments. Reasonable commitment to performance is the basis for corporate value added. At the same time, it provides reference for regulators to improve the merger and reorganization policy, the conditions for the use of performance commitments, and investors to rationally view the performance commitment system and make reasonable investment decisions.

## Supporting information

S1 Appendix Variable definition. (DOCX)
