# Supplementary material for: The effect of auditor’s cooperation with independent financial advisors on the reliability of performance commitment—Based on the moderating effect of managerial overconfidence
Source: PLoS One. 2023 Oct 26;18(10):e0283125. doi: 10.1371/journal.pone.0283125 (PMC10602356; doi:10.1371/journal.pone.0283125)
Supplement: S1 Appendix — (DOCX) [file pone.0283125.s001.docx]

**Appendix A. Variable Definition**

| **Variable** | **Definitions** |
| --- | --- |
| Variables of Interest |  |
| RVAM | During the performance commitment period, if the actual profit of each fiscal year is greater than or equal to the promised profit, it is 1; otherwise, it is 0. |
| Coop1 | Before this M & A transaction, the auditor has a cooperation experience with an independent financial adviser, which is 1, otherwise it is 0. |
| Coop2 | The natural logarithm of the number of auditors working with the IFA plus 1 |
| OC | The ratio of the sum of the top three executive salaries to the sum of all executive salaries, if it is greater than the median, it is defined as 1, otherwise it is 0 |
| Control variables |  |
| Lev | T -1 year ending total liabilities/ending total assets |
| ROE | Net profit/Stockholders' equity balance |
| Ownership | Defined as 1 when the acquirer is state-owned, otherwise 0 |
| Tobin Q | Market capitalization in year T-1 / Total ending assets |
| Institute | Number of shares held by institutional investors/total share capital |
| Growth | Acquirer's operating income growth rate |
| EC | The sum of the squares of the shareholding ratios of the top five major shareholders of the company |
| MB | The ratio of book value to the market value of the acquirer |
| Relate | Assigned to 1 for related transactions, 0 otherwise |
| DVAM | The value of the two-way performance commitment compensation agreement is 1, otherwise it is 0 |
| TVAM | Equity compensation and mixed compensation take the value of 1, otherwise it is 0 |
